# The breeding biology of erect-crested penguins, *Eudyptes sclateri*: Hormones, behavior, obligate brood reduction and conservation

**DOI:** 10.1371/journal.pone.0275106

**Published:** 2022-10-12

**Authors:** Lloyd S. Davis, Martin Renner, David Houston, Lei Zhu, Wiebke Finkler, Thomas Mattern

**Affiliations:** 1 Department of Science Communication, University of Otago, Dunedin, New Zealand; 2 Department of Zoology, University of Otago, Dunedin, New Zealand; 3 Department of Conservation, Wellington, New Zealand; 4 Department of Marketing, University of Otago, Dunedin, New Zealand; 5 The Tawaki Trust, Dunedin, New Zealand; 6 Global Penguin Society, Puerto Madryn, Argentina; MARE – Marine and Environmental Sciences Centre, PORTUGAL

## Abstract

Erect-crested penguins are the least studied of all penguins. They breed on two isolated subantarctic island groups, the Antipodes and Bounty Islands. Sporadic nest counts indicate a dramatic decline in numbers of erect-crested penguins over the last 50 years. Here we present data from a study undertaken in 1998 on the breeding biology, behavior and hormones of erect-crested penguins. It represents, even today, by far the most detailed data available on this species. The penguins exhibited extreme reversed egg-size dimorphism, whereby the first-laid A-egg was much smaller than the second-laid B-egg. A-eggs were lost before (42.3%) or on (37.8%) the day the B-egg was laid, and none survived more than 7 days after that. The penguins were in a low state of reproductive readiness, as evidenced by low levels of copulation, fighting, and testosterone in males during the courtship/laying period when, curiously, plasma levels of testosterone were at least as high in females. The laying interval (5.4 days) is the longest recorded for any penguin species, and incubation was highly variable until clutch completion. Most nests (91.2%) contained no nesting material and eggs were laid directly onto the ground. A-eggs were lost mainly by rolling out of the nest. However, even when prevented from doing so by an experimental manipulation, A-eggs survived no longer than those in control nests. Testosterone levels in males increased after clutch completion, when they remained in attendance at the nest for up to 13 days, despite females assuming most of the incubation duties. The bills of males were significantly larger than those of females and probably help with guarding the nest. We discuss explanations for obligate brood reduction in crested penguins and the options for conservation in light of our census results, which indicate that this enigmatic penguin species could be in trouble.

## Introduction

When three of us (LSD, MR and DH) went to the Antipodes Islands in 1998 to conduct research on the erect-crested penguin (*Eudyptes sclateri*), it was by far the world’s least studied penguin species [1]. Twenty-four years on, sadly, the situation remains unchanged: the very few studies conducted since then have been involved mostly with just counting the numbers of erect-crested penguins breeding on the Bounty and Antipodes Islands south-east of New Zealand. These studies have documented further drastic declines in the population of erect-crested penguins on the Antipodes Islands [2, 3] from those that we had observed in 1998 [4, 5]. Findings from our 1998 research were presented in papers at three conferences [6–8] and a summary derived from our data is given in Davis [5]. However, our observations from 1998 remain the most recent and most extensive data on aspects of the breeding biology and behavior of erect-crested penguins. Together with our other co-authors, we have here compiled and analyzed those data comprehensively so that they may be used as reference points for future efforts to address the apparent decline in erect-crested penguins and to explore the biology of this enigmatic penguin species.

### The erect-crested penguin

The erect-crested penguin belongs to the genus *Eudyptes*, the crested penguins, which consist of seven species that are distinguished by having prominent yellow or orange crests above their eyes. In the case of the erect-crested penguin, the yellow feathers above each eye form a distinctive upright crest [9]. Crested penguins are also characterized by their extreme intra-clutch egg-size dimorphism [10, 11], although the erect-crested penguin remains the least studied of the *Eudyptes* species in this regard [12].

Erect-crested penguins breed mainly on the Antipodes and Bounty Islands from September to late January and remain offshore between breeding seasons [5]. Clutches consist largely of two eggs, with the first egg (A-egg) being laid in early October and the larger second egg (B-egg) being laid approximately five days later. Some observations suggest that the A-egg may be broken, rolled away or even deliberately ejected by the female, with typically only the B-egg being incubated [12–14].

There have never been any full-season observations at the breeding colonies of erect-crested penguins. Systematic research on this species is very limited. Richdale [15] recorded the breeding of an aberrant single pair of erect-crested penguins that bred on the Otago Peninsula from 1938 to 1940. Warham [16] deduced the timing of breeding, and described the nest sites and characteristic of chicks based upon observations at the Antipodes Islands from late January to March (i.e., the late breeding season) in 1969. In November 1978, researchers spent two weeks at the Bounty Islands and described the local population, incubation behaviors, daily activities, as well as the egg-size dimorphism of erect-crested penguins [13]. Further notes on egg-size dimorphism and causes of egg losses were made during a short visit to the Antipodes Island in October 1990 [14]. There have also been a few short-term surveys on the Bounty and Antipodes Islands focused on censusing the local population of breeding penguins [2, 17, 18].

### Egg size dimorphism and obligatory brood reduction

Birds often face limited amounts of available natural resources, especially with regard to the availability and distribution of food. In particular, when breeding, parent birds must acquire enough food to meet the energy requirements for breeding while also allocating sufficient resources to their chicks [19]. A prominent clue to the trade-offs involved in such allocations is revealed by the degree of intraspecific variation in egg sizes [19–21]. Such variations are important determinants of avian reproductive success because egg sizes affect chick sizes, which has a major influence on the quality and survival rate of offspring [22, 23]. Smaller chicks within a clutch are more likely to starve, suggesting a positive relationship between resource allocation and reproductive success [24, 25]. Variation of intraspecific egg sizes reflects how females with different phenotypes and niches allocate their investments [20]. There are two contributors to intraspecific egg size variation: (i) that resulting from inter-clutch egg size variation, and (ii) that resulting from intra-clutch egg size variation.

Christians [20] suggested that inter-clutch egg size variation makes up approximately 70% of intraspecific avian egg size variation. By contrast, usually the degree of egg size variation within each clutch is much less extreme, making up only 9%– 30% of the total intraspecific variation in egg size [22, 26].

The most extreme variation in egg size of all birds is reported in the crested penguins [27]. All seven species of the crested penguins lay a clutch of two eggs. The A-egg is significantly smaller than the B-egg, with the difference in size ranging from 25% to 70% [28, 29]. Such extreme egg-size dimorphism facilitates asynchronous incubation and hatching, and is associated with obligate brood reduction. In those *Eudyptes* penguins that have relatively modest egg size dimorphism, such as Fiordland penguins (*Eudyptes pachyrhynchus*) and Snares penguins (*Eudyptes robustus*), both the A-egg and B-egg are typically incubated, with 31% - 60% of nests hatching both chicks. However, in most cases, only a single chick is fledged [10, 12]. By contrast, in those *Eudyptes* penguins with the most extreme egg-size dimorphism, such as erect-crested penguins and Macaroni penguins (*Eudyptes chrysolophus*), one egg–typically the A-egg–is lost and only one chick hatches [12, 27, 29].

Researchers have paid increasing attention to such an enigmatic trait of *Eudyptes* penguins: how and why the A-egg is lost and what might be the function of the smaller A-egg [11, 27, 29, 30]? Massaro et al. [30] observed that the survival of A-eggs in nests of Snares penguins was influenced by the aggressive behaviors of male penguins.

An early hypothesis to explain the function of the smaller egg suggested that it may be an insurance against the possible loss of an egg [10, 31]. However, in most other species of birds that lay an extra egg as an insurance against egg loss–using egg-size dimorphism to facilitate brood reduction if the loss does not occur–the extra smaller egg is usually the last-laid egg, whereas the crested penguins have reversed egg-size dimorphism, with the first-laid egg being smaller [11, 32]. Moreover, in some *Eudyptes* species, such as Macaroni and royal penguins (*Eudyptes schlegeli*), most A-eggs have already been lost before B-eggs are laid and the females do not start incubation until after the B-egg is laid, suggesting that the A-egg is an unlikely form of insurance against loss of the larger B-egg [33].

Another hypothesis suggested that the A-egg may provide stimulation for forming the brood patch [33]. While this was supported by a cross-fostering experiment in Fiordland crested penguins [33], a later study on southern rockhopper penguins (*Eudyptes chrysocome*) did not find evidence to support this hypothesis [27]. Other hypotheses for the function of the smaller A-eggs in crested penguins include suggestions that it reduces costs due to fighting during the courtship period, functions as a visual signal of occupied nest sites or facilitates laying synchrony within a colony–although these explanations have not been tested [11, 27].

Another hypothesis is that the small A-egg does not have a specific adaptive function but its size is a consequence of a physiological constraint (the migratory carry-over effect) [34]. When crested penguins are still at sea and returning to the breeding colony from their annual migration, females start to form the eggs. Those females arriving in the colony early will have a longer time between arrival and laying, and should tend to produce larger A-eggs. However, in a study on eastern rockhopper penguins (*Eudyptes chrysocome filholi*), Morrison [32] found that the pre-laying interval was not related to egg-size dimorphism, which did not support that the migratory carry-over effect hypothesis.

In summary, up till now, there has not been a widely accepted hypothesis for the egg-size dimorphism and obligatory brood reduction of crested penguins due to a lack of systematic data and relevant experimental tests in breeding colonies of different species of crested penguins, particularly those with extreme egg-size dimorphism [12, 27, 32].

In this paper, we present data from a study of erect-crested penguins carried out on the main Antipodes Island from 18 September to 2 November 1998, which covered the courtship and laying periods. We conducted a census of a subsample of colonies breeding on the island, and followed the fates of 270 banded individuals in a single study colony using 113 nest sites. We measured the degree of sexual dimorphism of males and females, recorded the dates of laying, plus the timing and causes of egg losses. Using penguins breeding in another colony, we sampled the degree of egg-size dimorphism, took blood samples to measure levels of testosterone and estrogens, and conducted an experiment to reduce losses of A-eggs by preventing them rolling away from the nest. Our aim here is to present information on the breeding biology of erect-crested penguins that may be used to foster understanding of the causes for their population declines and the reversed egg-size dimorphism and obligatory brood reduction characteristic of these penguins.

## Methods

### Study site

We studied erect-crested penguins breeding on Antipodes Island, which is the largest island of the Antipodes Islands archipelago some 860 kilometers south east of New Zealand, from 18 September– 2 November 1998.

### Study Colony and behavioral observations

The main study colony (hereafter termed, Study Colony) was atop a relatively flat rocky platform in Anchorage Bay (49°39’57”S, 178°47’59”E). It was chosen because all nests in the colony could be observed simultaneously from an observation platform on the adjacent cliff face.

Before any egg-laying began in the Study Colony, 270 erect-crested penguins were caught by placing a net on a long pole over them. They were flipper banded, weighed with a Persola balance (±5g), and morphometric measurements were taken of their bill length, bill depth, flipper length, foot length and crest length using calipers (± 0.05mm). The birds had a unique code of a letter and a number painted on their backs in yellow enamel paint so that they were individually identifiable from the observation platform. Thereafter, the Study Colony birds were not handled again in any way and the colony was not entered again by us.

We observed the Study Colony for up to 12 hours/day, for a total of 249 observation hours, from 29 September to 22 October. This coincided with the courtship, laying and immediate post-laying periods. We used all-occurrences sampling [35] to record the time and identities of participants in all instances of copulating and fighting throughout the Study Colony. Additionally, we used instantaneous scan sampling [35] to record the behaviors every 15 minutes of individuals present within a subsample of 19 nests. The 113 nests making up the Study Colony were mapped and we noted the identities of birds attending each nest, the presence of nesting material, the dates on which the A- and B-eggs were laid, as well as the dates and causes of any egg losses.

Sizes of 22 A-eggs were measured using calipers (± 0.05mm) and weighed (±0.25g) from a sample of nests in the Study Colony when the A-egg had been rolled out of the nest and we were able to collect the egg without entering the colony or displacing the nesting birds. Laying dates were known for 16 of the eggs. Sizes of 51 B-eggs were measured similarly at the conclusion of the study, of which the laying date was known for 49. A pole was used to gently leverage the rear end of the bird so the B-egg could be removed and measured quickly. The whole procedure took about a minute and, when the egg was replaced in the nest, all birds resumed incubation.

Discriminant function analysis (DFA) is a statistical technique used to make decisions about naturally occurring group membership, such as sex. It is a commonly used method used to sex penguins, which are monomorphic in appearance, whereby a function combining morphometric measurements such as bill length, bill depth, flipper length or foot length is determined that best predicts the sex of a bird accurately [36–38]. We calculated the best discriminant function using morphometric measurements from a sample of penguins for which we knew their sex unequivocally (determined separately using behavioral observations).

### Experimental colony

A sample of 42 nests of erect-crested penguins breeding in a nearby colony, also in Anchorage Bay, was used for an experimental manipulation. A ring of stones was placed around 14 nests (Experimental Group) to prevent eggs being rolled away from the vicinity of the nests to determine whether this could influence retention and survival of the first-laid A-egg. There were 28 nearby nests in the colony were monitored as a Control Group. The 42 nests were inspected daily to record the dates that A-eggs and B-eggs were laid. A chinagraph pencil was used to mark the A- and B-eggs for identification. The dates of egg losses, fates of lost eggs, and the date that the female began the first incubation spell (i.e., the date on which the male had departed for sea) were recorded.

### Hormones

Blood samples (1ml) were taken from the brachial vein of penguins breeding in nests elsewhere in Anchorage Bay during egg-laying (n = 5 males; 5 females), the post-laying guard period (n = 6 males; 6 females), and the first incubation spell (n = 6 females). Following that, morphometric measurements of the penguins (bill depth, bill length) were taken, as described above, which were used in combination with behavioral observations to determine their sex. Measurements and blood sampling were completed within 5 minutes for each bird. Individual birds were sampled only once.

Plasma was separated from blood by centrifugation within 1 hour of bleeding and immediately frozen by storing in liquid nitrogen. Upon return from the Antipodes, samples were kept at -80°C until analyzed within the next 12 months. Plasma concentrations of the sex steroids estradiol and testosterone were measured by specific radio-immunoassays. The sex steroids were extracted in diethyl ether and assayed using the methods of [39–41]. Extraction efficiency was > 90% for all steroids (determined by recovery of ^3^H-steroids added to plasma samples) and values were not corrected for recovery. Percentage cross reactions as supplied by the antisera manufacturer (Endocrine Sciences, Tarzana, CA) were as follows: Estradiol: estrone, 1.3; Estriol, 0.6; 19 other steroids, < 0.05. Testosterone: dihydrotestosterone, 44; delta-1-testosterone, 41; delta-1-dihydrotestosterone, 5α-androstan-3β, 17β-diol, 3.0; 4-androstan-3β, 17β-diol, 2.5; delta-4-androstenedione, 1.5; 24 other steroids, < 0.5.

### Erect-crested penguin census

On 27 and 28 October, we carried out a census of erect-crested penguins breeding at five locations on Antipodes Island (Alert Bay North, Orde Lees, Anchorage Bay, Stella Bay and Reef Point). The locations were suggested by the Department of Conservation in order to be comparable with previous counts made on Antipodes Island. Three of us (LSD, MR, DH) each counted the number of active nests of breeding birds using hand counters and the counts were averaged. Orde Lees was difficult to count accurately due to the large number of birds present and our limited access, which meant that the penguins had to be counted from a distance of over 100 meters using binoculars. As an alternative to using the hand counters, each of us also counted the number of sections containing 70 birds that fit within the colony.

Data were analyzed using MS Excel and SPSS Version 24.0. Permits for landing on Antipodes Island and handling the erect-crested penguins were issued by New Zealand’s Department of Conservation (permit #s 98-99/443/01/07, 98-99/443/10/03, and 98-99/443/05/01).

## Results

### Morphometric measurements and sexual dimorphism

In all, we banded 270 individual penguins in the Study Colony from 20 September to 30 September, which included 136 males, 121 females and 13 for which we did not have any corroborating evidence to assign a sex. The sex of individual penguins was verified by either observation of copulatory position, tread marks on the back (indicating a female), performance of ecstatic displays during the courtship period (indicating a male), or a combination of these. Males and females were sexually dimorphic, with male penguins being significantly larger than females in all morphometric measurements except crest length (Table 1). Furthermore, in all nests where behavioral data could be used to determine sex, the male was always the larger of a pair. Sexual dimorphism is most pronounced in the bill, with males having a noticeably more robust bill that is deeper and longer than that of females.

**Table 1:** Results of morphometric measurements from 121 females and 136 males.

	Females (n = 121)	Males (n = 136)
Weight (kg)**	5.11 ± 0.35	5.24 ± 0.35
Bill length (mm)**	54.68 ± 2.08	60.05 ± 2.30
Bill depth (mm)**	22.66 ± 0.89	26.14 ± 1.10
Foot length (mm)**	118.68 ± 3.71	123.92 ± 3.47
Flipper (mm)**	163.88 ± 4.15	171.15 ± 5.11
Crest (mm)	53.04 ± 3.24	52.94 ± 3.44

Measurements are given with standard deviations (SD).

** indicates a significant difference between females and males (*t*-test, weight: *t*(255) = -2.98, P = 0.003; bill length: *t*(255) = -19.55, P < 0.001; bill depth: *t*(255) = -27.65, P < 0.001; foot length: *t*(255) = -11.70, P < 0.001; flipper: *t*(255) = -12.43, P < 0.001); no asterisk indicates no significant difference between sexes (crest: *t*(255) = 0.24, P = 0.81).

Discriminant function analysis (DFA) showed that using bill depth and bill length allowed assignment of sex with 96.9% accuracy in the Study Colony. The discriminant function indicates that bill depth is the most important variable to discriminate between sexes (Wilks’ Lambda = 0.250, F = 764.700, P < 0.001), followed by the bill length (Wilks’ Lambda = 0.400, F = 382.181, P < 0.001; NB: a smaller value of Wilks’ Lambda represents a better predictor). Calculating a Bill Index by multiplying bill depth (mm) × bill length (mm) proved to be the best predictor in the discriminant model for assigning sex in erect-crested penguins (Wilks’ Lambda = 0.231, F = 846.875, P < 0.001, overall accuracy = 96.9%). As a simple rule, sex can be assigned with reasonable confidence if the Bill Index is under 1331.0 (probability of female = 95.0%) or over 1478.5 (probability of male = 95.0%). Using just this simple rule accurately identifies the sex of 86.3% of the birds in our Study Colony, with only 13.7% of birds falling within the overlap range.

### Breeding and egg size dimorphism

**Fig 1:**
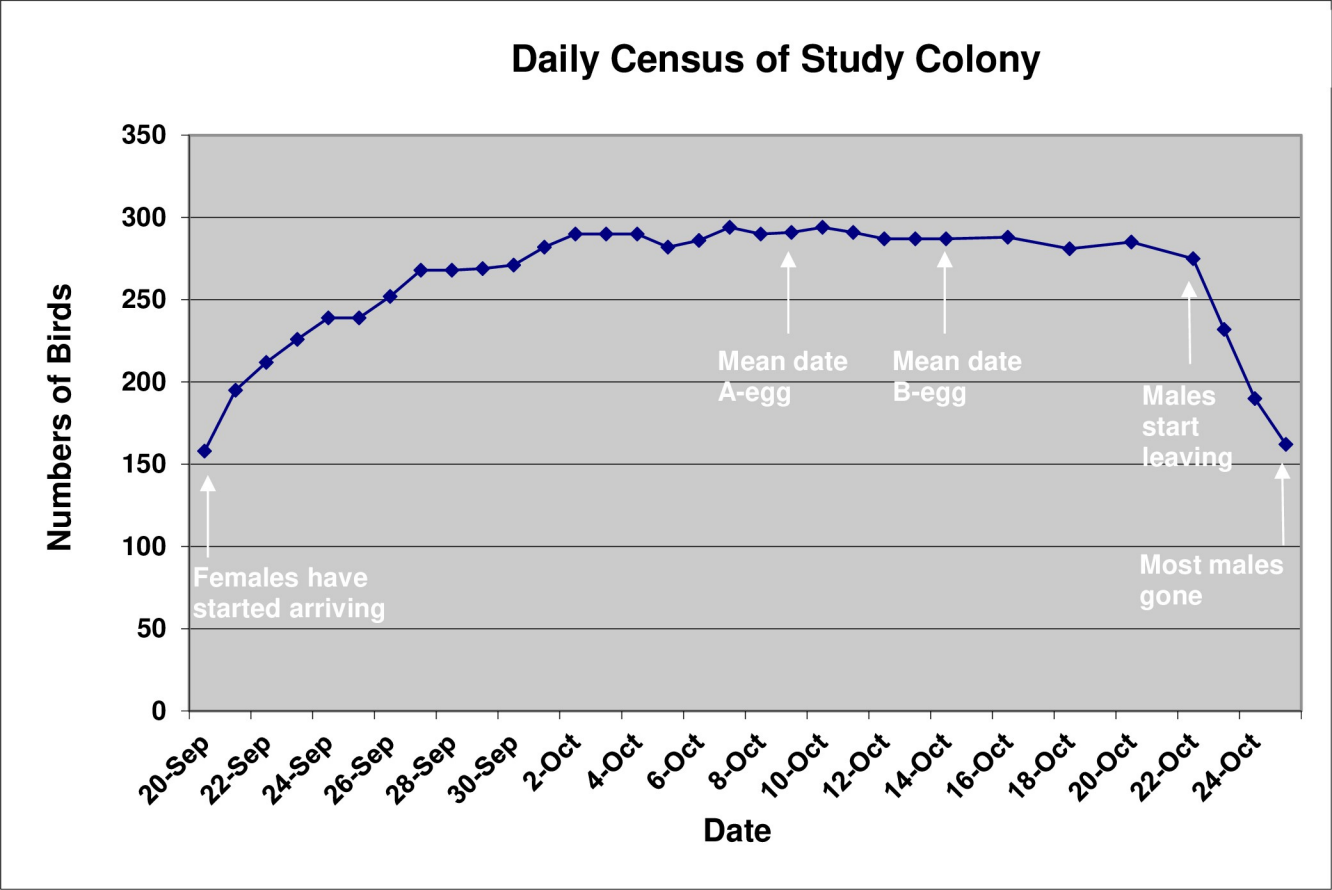
Daily census of number of erect-crested penguins in the Study Colony.

The majority of the 158 penguins present in the Study Colony when we began our observations and banding on 20 September were males. Of the 24 birds that we caught and banded that day, 19 (79.2%) were males and only 5 (20.8%) were females. If that ratio was a representative sample, then the colony would have contained around 125 males at the start of this study. Thereafter, numbers of birds in the colony increased steadily, especially due to an influx of females, reaching 290 by 2 October and staying at 290 ± 3 until 16 October (Fig 1).

The nests of the erect-crested penguins were extremely simple: of 113 nests in the Study Colony, 103 (91.2%) did not use any nesting material at all, with the birds laying their eggs on the rocks or bare ground. Only 10 (8.8%) nests contained any nesting material and this consisted mostly of straw-like dried grasses.

During the courtship period, males lost body mass at a significantly greater rate (63.8g/day) than did females (53.6g/day) (P<0.05). Males also tended (P = 0.06) to spend a higher percentage of their time fighting (0.25%) than did females (0.05%) during the courtship period, although in both sexes the amount of fighting was very low and fights were infrequently observed in the Study Colony. Copulation rates were also very low, with a mean of 0.03 copulation attempts per hour per pair.

**Fig 2:**
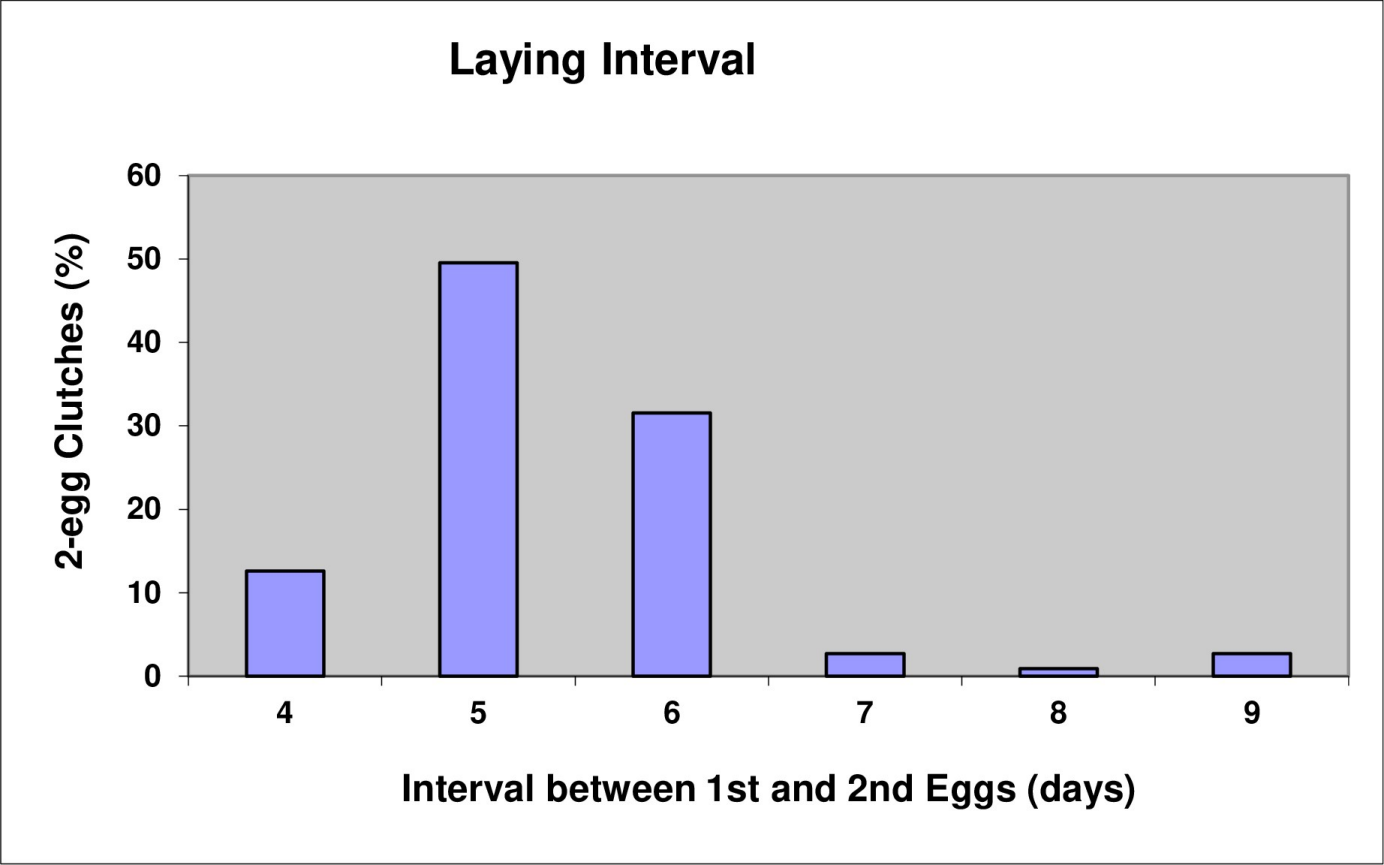
Laying intervals for two-egg clutches of erect-crested penguins (n = 111).

The first A-egg was laid in the Study Colony on 3 October and the last clutch was initiated on 14 October. The mean date for laying A-eggs was 8 October (SD = 2.42, n = 118) and the mean laying date for B-eggs was 13 October (SD = 2.57, n = 112). The mean intra-clutch laying interval was 5.38 days (SD = 0.96, n = 111) with the range being 4–9 days. For 93.6% of nests the laying interval was 4–6 days (Fig 2). Furthermore, there was no difference in the mean laying interval for early breeding females (i.e., those laying A-eggs on or before the mean laying date of 8 October) (5.35 days, SD = 0.92, n = 62) compared to later breeding females (5.41 days, SD = 0.95, n = 49) (*t*-test, *t*(102) = 0.29, P = 0.77).

**Fig 3:**
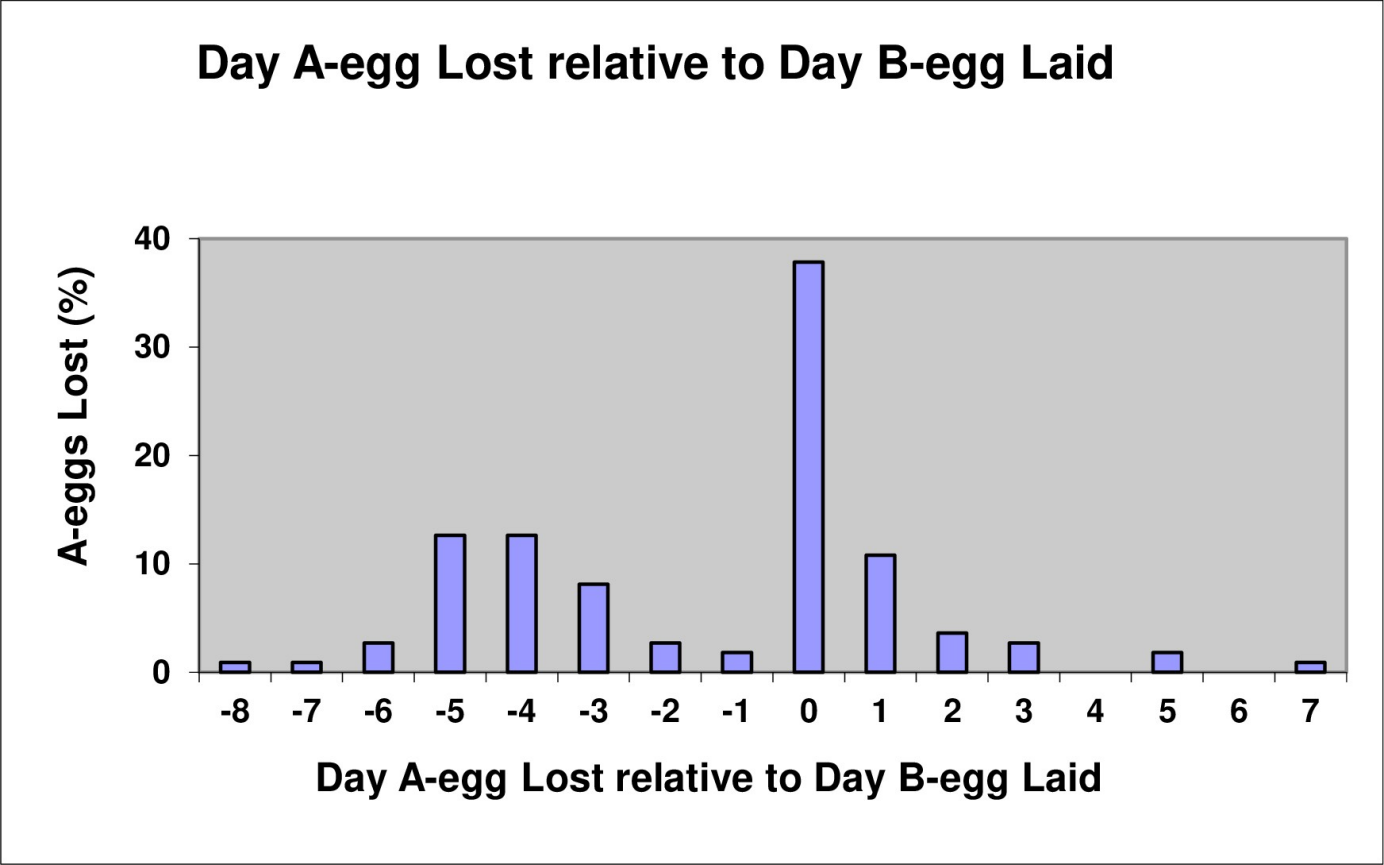
The day A-eggs were lost relative to the day of laying of the B-egg (n = 111).

Of the 111 A-eggs from two-egg clutches, none survived more than a week after the B-egg was laid, with the vast majority (80.1%) being lost before (42.3%) or on the day of the B-egg being laid (37.8%) (Fig 3).

**Fig 4:**
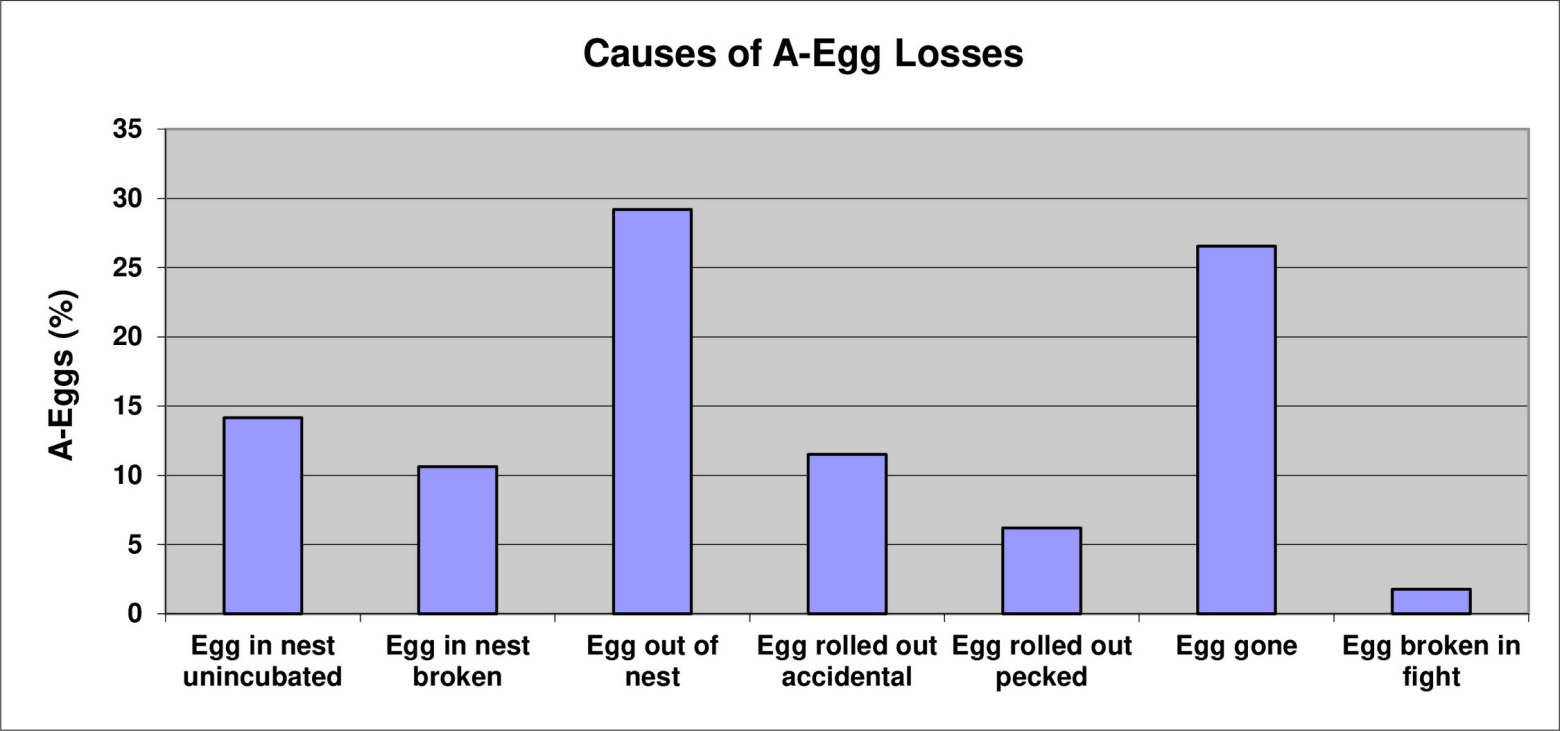
Causes of losses of A-eggs laid in the Study Colony (n = 113).

Of A-eggs monitored in 113 nests in the Study Colony, only about a quarter (24.8%) were lost within the nest, either through the parents ignoring the egg and not incubating it (14.2%) or breaking it (10.6%) (Fig 4).

For 29.2% of nests, the A-egg was observed outside the vicinity of the nest, while for another 26.5% of nests the A-egg disappeared from one observation period to the next, most likely from rolling off the slightly sloping rocky platform on which the colony was sited or being taken by skuas once the egg was outside the nest. We observed two means by which the eggs ended up out of the nest: in 13 nests (11.1%), the A-egg was observed being rolled from the nest, seemingly accidentally, as a consequence of the incubating parent shuffling about on the nest while trying to adjust the eggs against its brood patch. This occurred especially on the day the B-egg was laid, as the birds tried to accommodate both the large B-egg and much smaller A-egg. In 7 instances (6.2%), we observed a parent bird peck at the A-egg, rolling it away from the nest. Fights were remarkably rare compared to other penguins during the courtship and laying period (see below), and in only 2 instances (1.8%) were A-eggs broken as a consequence of fighting in the colony (Fig 4).

**Fig 5:**
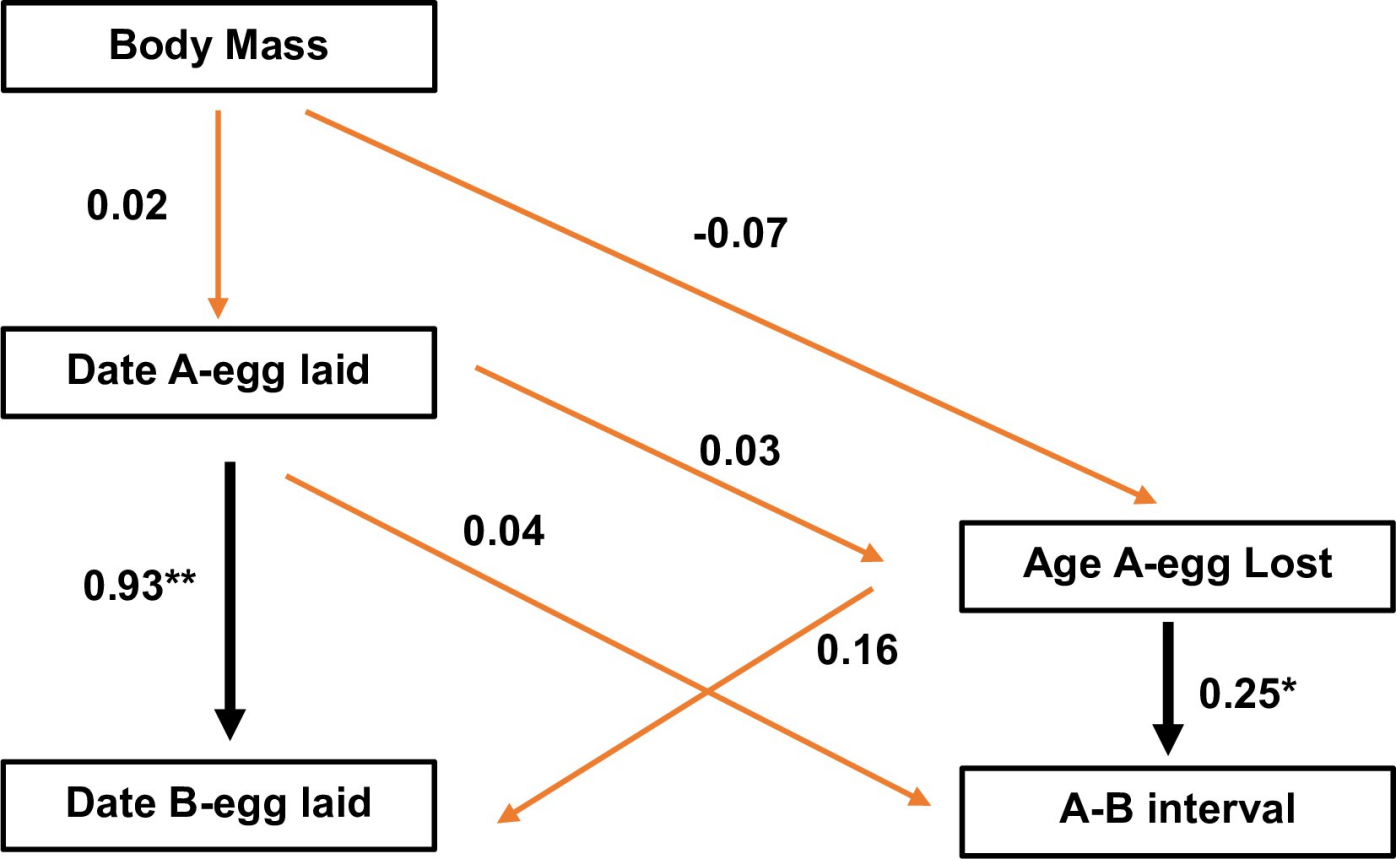
Correlations between the body mass of the female, dates of laying for A- and B-eggs, the laying interval (A-B interval) and the age at which the A-egg was lost. Significant correlations are shown with bold lines (** P < 0.001; * P < 0.05).

Correlations of the age at which the A-egg was lost with body mass of the female and the dates of laying the A- and B-eggs are presented in Fig 5. Body mass of the penguins did not affect either the timing of breeding or the age at which the A-egg was lost. As might be expected given the similarities in laying intervals (Fig 1), the date the B-egg was laid was highly correlated with the date the A-egg was laid (Pearson’s *r*(109) = 0.93, P < 0.001). Where the laying interval was longest, A-eggs tended to survive for longer (Pearson’s *r*(109) = 0.25, P = 0.009). This accords with the observed heightened vulnerability of A-eggs on the day that the B-egg is laid: the presence of the B-egg hastens the demise of the A-egg, often because parents have difficulty accommodating the two extremely dimorphic eggs in their brood patch. Parents were observed to push the large B-egg preferentially into the back position of their brood patch while then trying to accommodate the smaller A-egg in front. The A-egg was more inclined to roll away from that position if the incubating bird sat up and, if so, may even be pecked at by a parent.

The intra-clutch egg size dimorphism was extreme (Table 2). On average, the B-egg (n = 51) is 85% heavier than the A-egg (n = 20) (mass A/B = 0.54). In terms of egg volume, the B-egg is 79% larger than A-egg (n = 22) (volume A/B = 0.56).

**Table 2:** Comparison of A-eggs (n = 22) and B-eggs (n = 51) of erect-crested penguins.

	A-egg	B-egg
Length (mm)**	69.17 ± 3.75	83.84 ± 3.28
Breadth (mm)**	46.41 ± 1.68	57.55 ± 2.05
Volume (mm^3^)**	77987.73 ± 7870.17	139631.44 ± 12651.83
Mass^1^ (g)**	81.55 ± 7.56	150.94 ± 17.92

The volume was calculated based on length and breadth, according to Narushin [66]. *t*-tests were applied to identify the significance of differences between A-egg and B-egg: ** means the difference was significant (*t*-test, length: *t*(71) = -16.78, P < 0.001; breadth: *t*(71) = -22.43, P < 0.001; volume: *t*(71) = -21.11, P < 0.001; mass: *t*(69) = -16.69, P < 0.001).

^1^Mass was available for n = 20 A-eggs.

**Fig 6:**
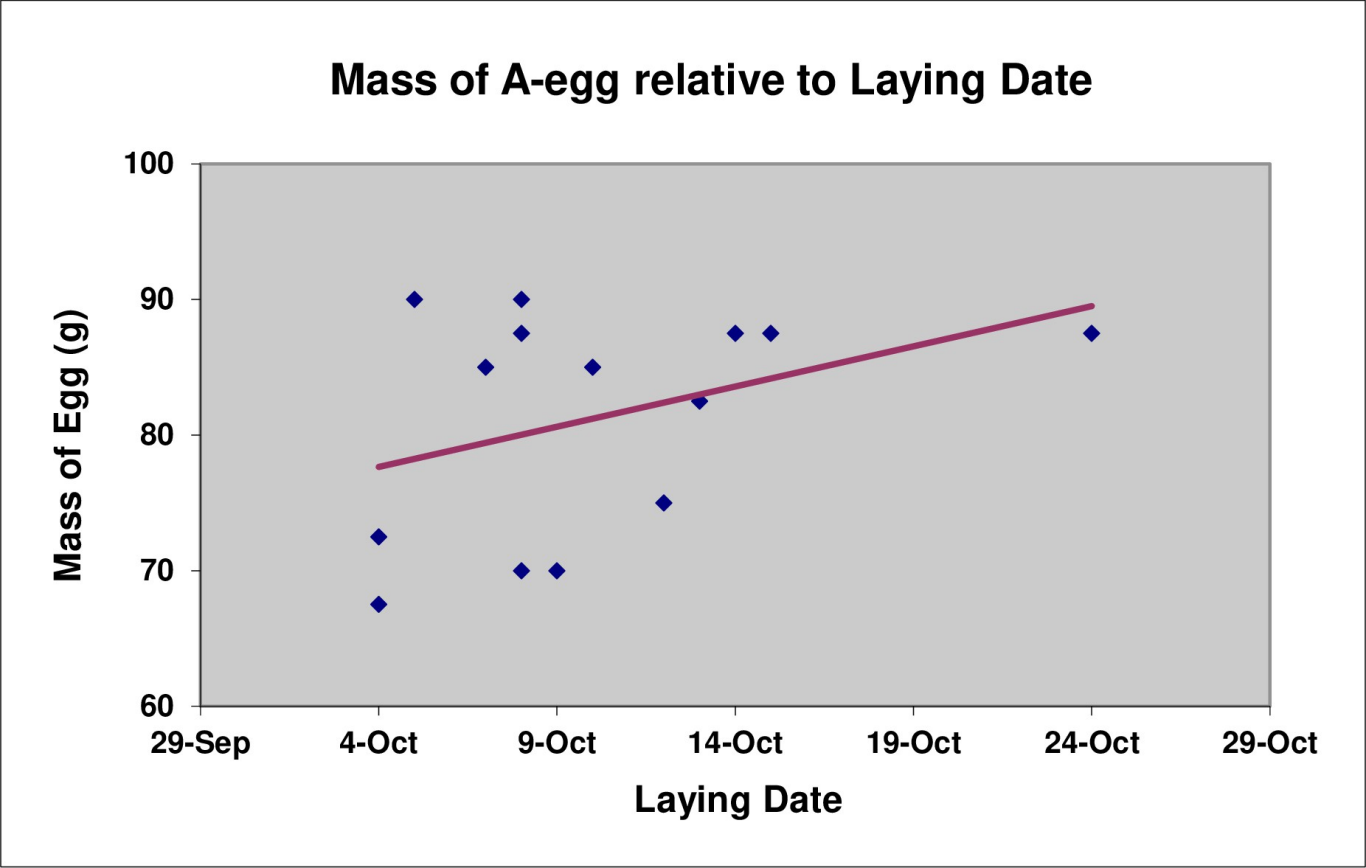
Mass (g) of A-eggs relative to laying date (n = 14).

**Fig 7:**
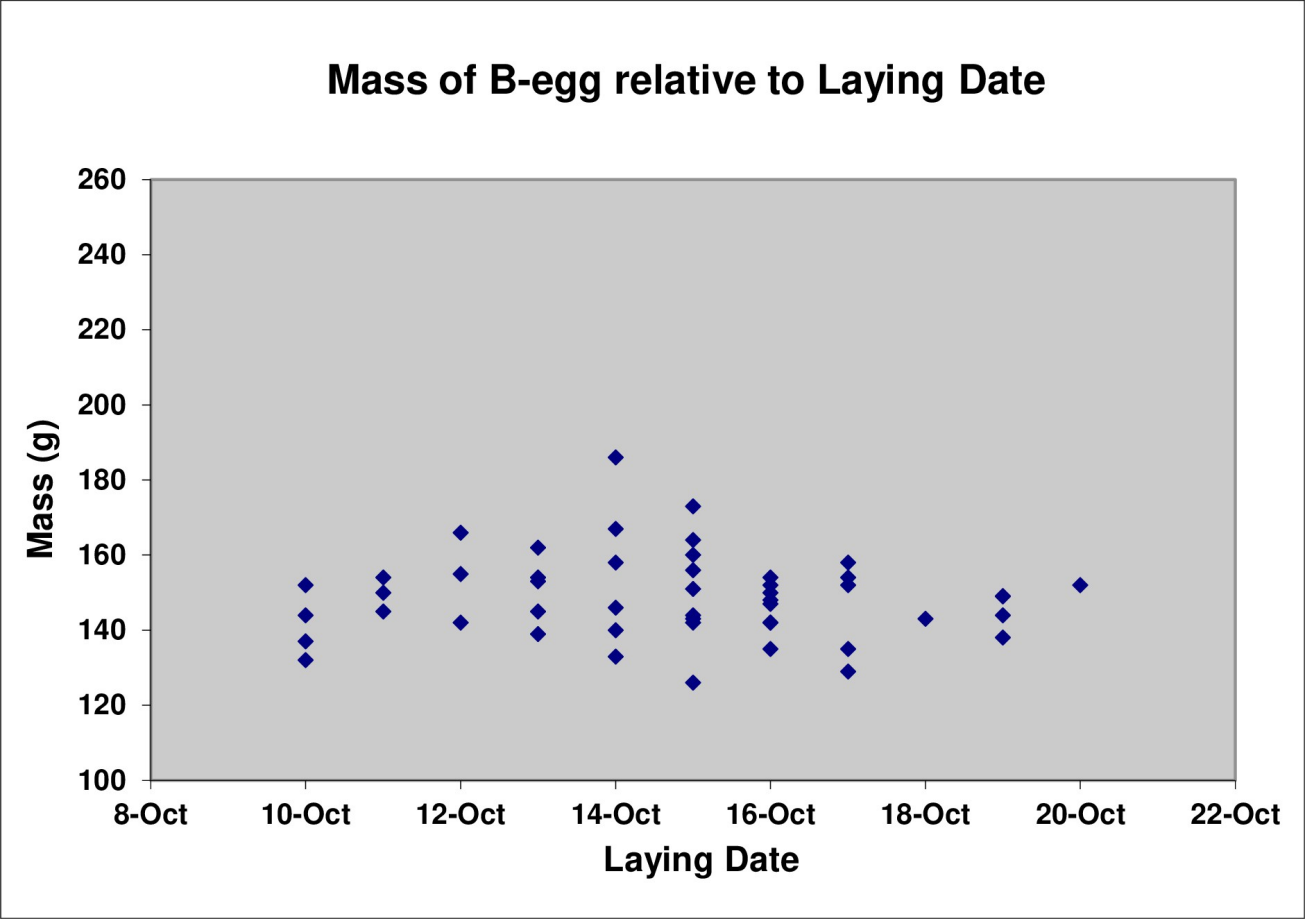
Mass of B-eggs relative to laying date (n = 49).

There is the suggestion of a tendency for the mass of A-eggs to increase with laying date (Pearson’s *r*(12) = 0.38, P = 0.18) (Fig 6), although the sample size of A-eggs for which we had both the mass of the egg and its laying date was quite small (n = 14). By contrast, the mass of B-eggs did not change significantly with laying date (Pearson’s *r*(41) = 0.04, P = 0.98) (Fig 7).

### Incubation behavior

**Fig 8:**
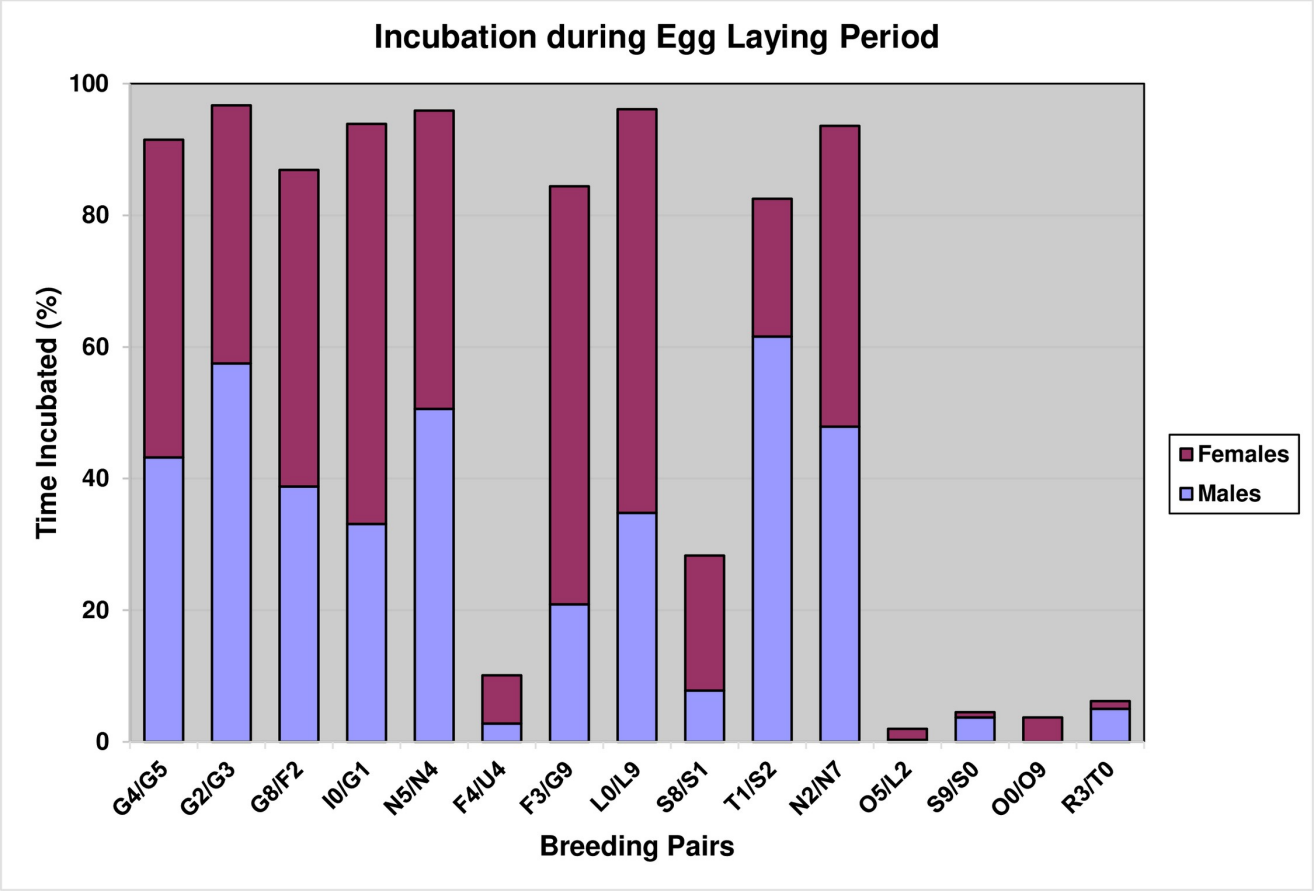
Percentage of time males and females in 15 nests spent incubating the A-egg prior to laying of the B-egg.

**Fig 9:**
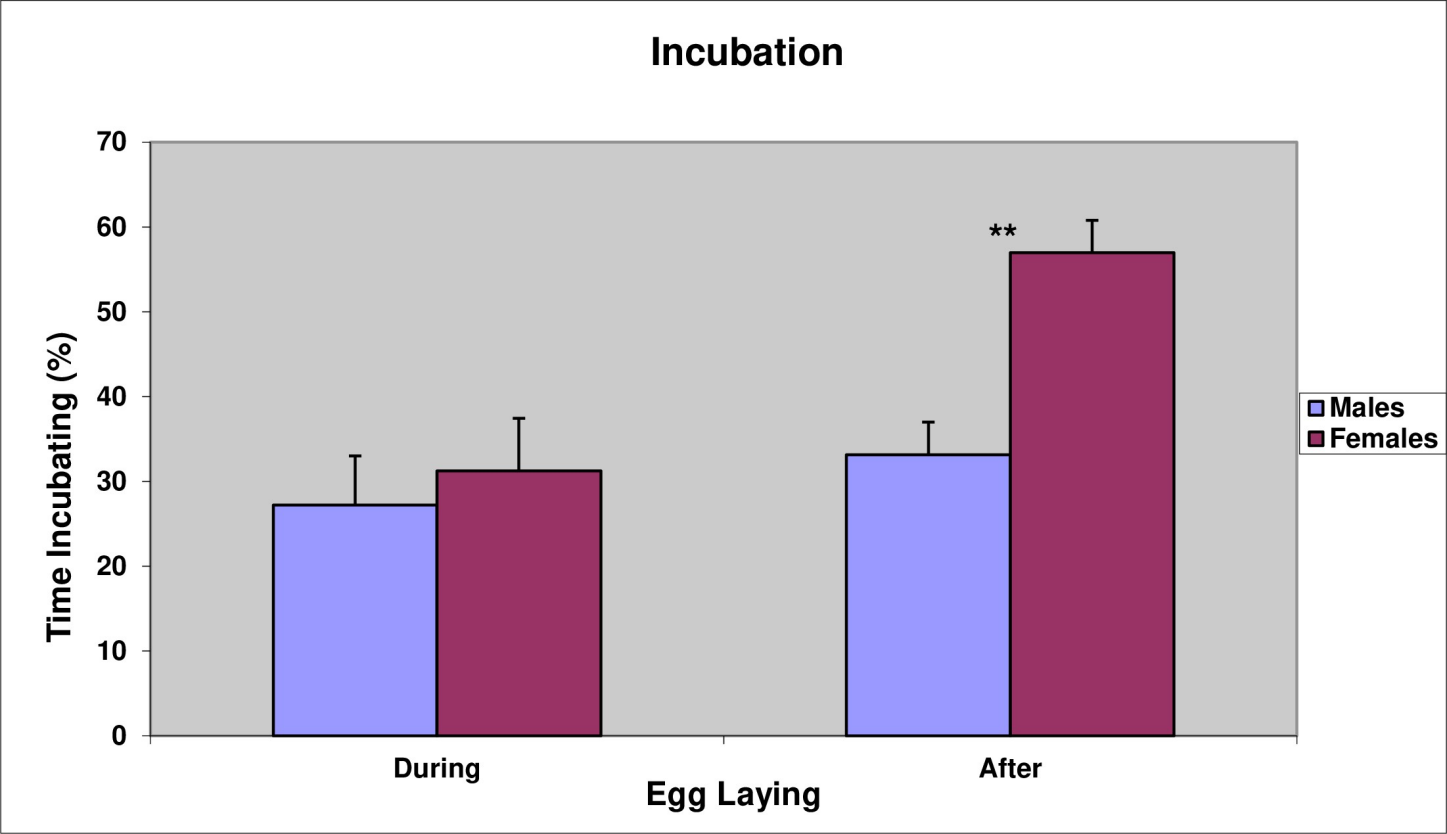
Mean percentage of time males and females of 15 pairs spent incubating prior to laying of the B-egg (i.e., during egg laying) and afterwards. ** indicates that the difference between males and females is highly significant (P < 0.01).

Instantaneous scan sampling revealed that between the laying of the A-egg and B-egg, 40% of pairs largely neglected the A-egg and did very little incubation (Fig 8), with those pairs that did incubate, sharing the incubation duties more or less equally between the male and female (Paired *t*-test, *t*(14) = 0.79, P = 0.44). Whereas, following the laying of the B-egg, the female took the dominant role in incubating the egg (Fig 9, Paired *t*-test, *t*(14) = 3.14, P < 0.01).

### Experiment to prevent eggs being rolled away

**Fig 10:**
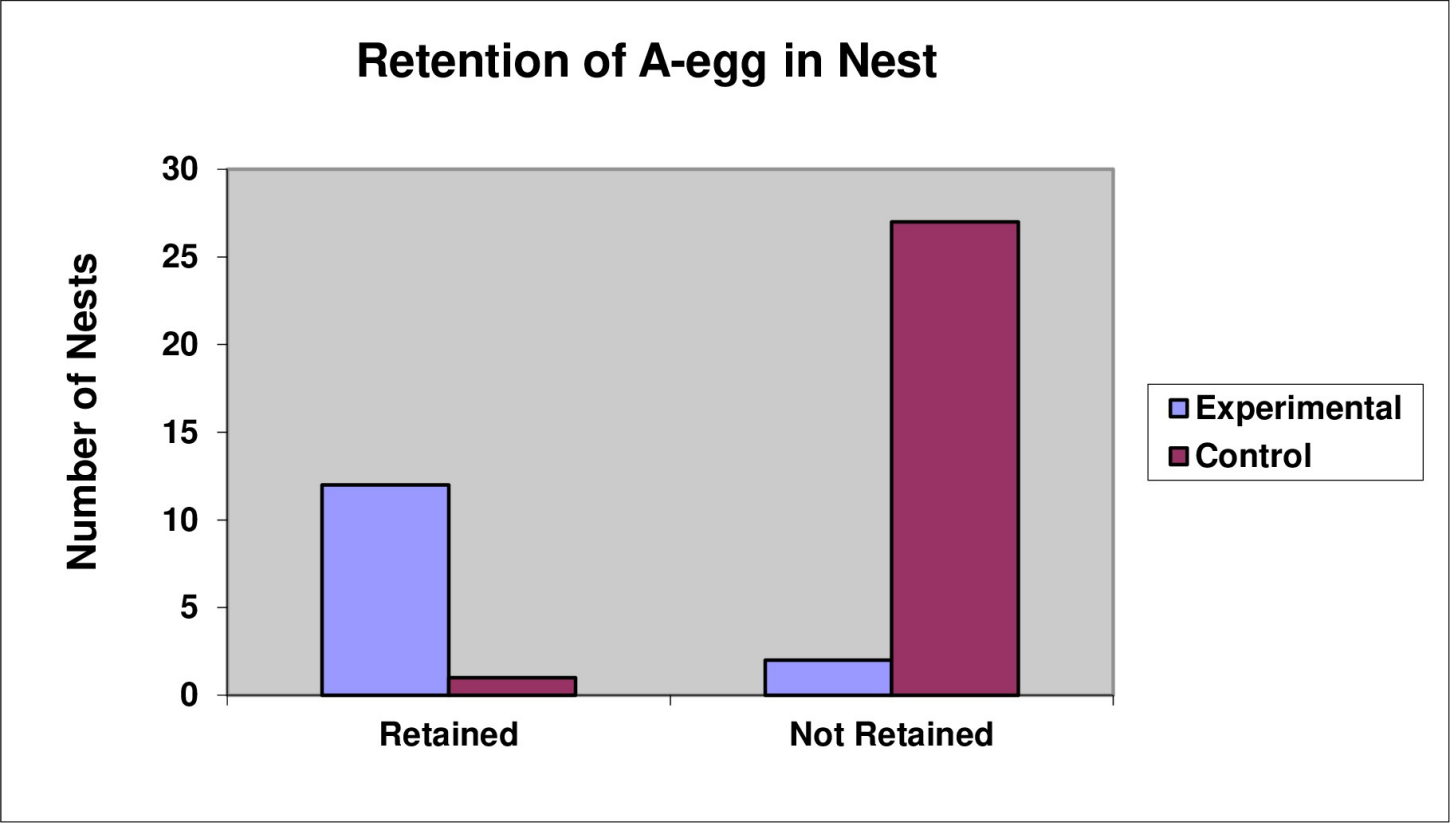
Retention of A-eggs in Experimental and Control nests.

**Fig 11:**
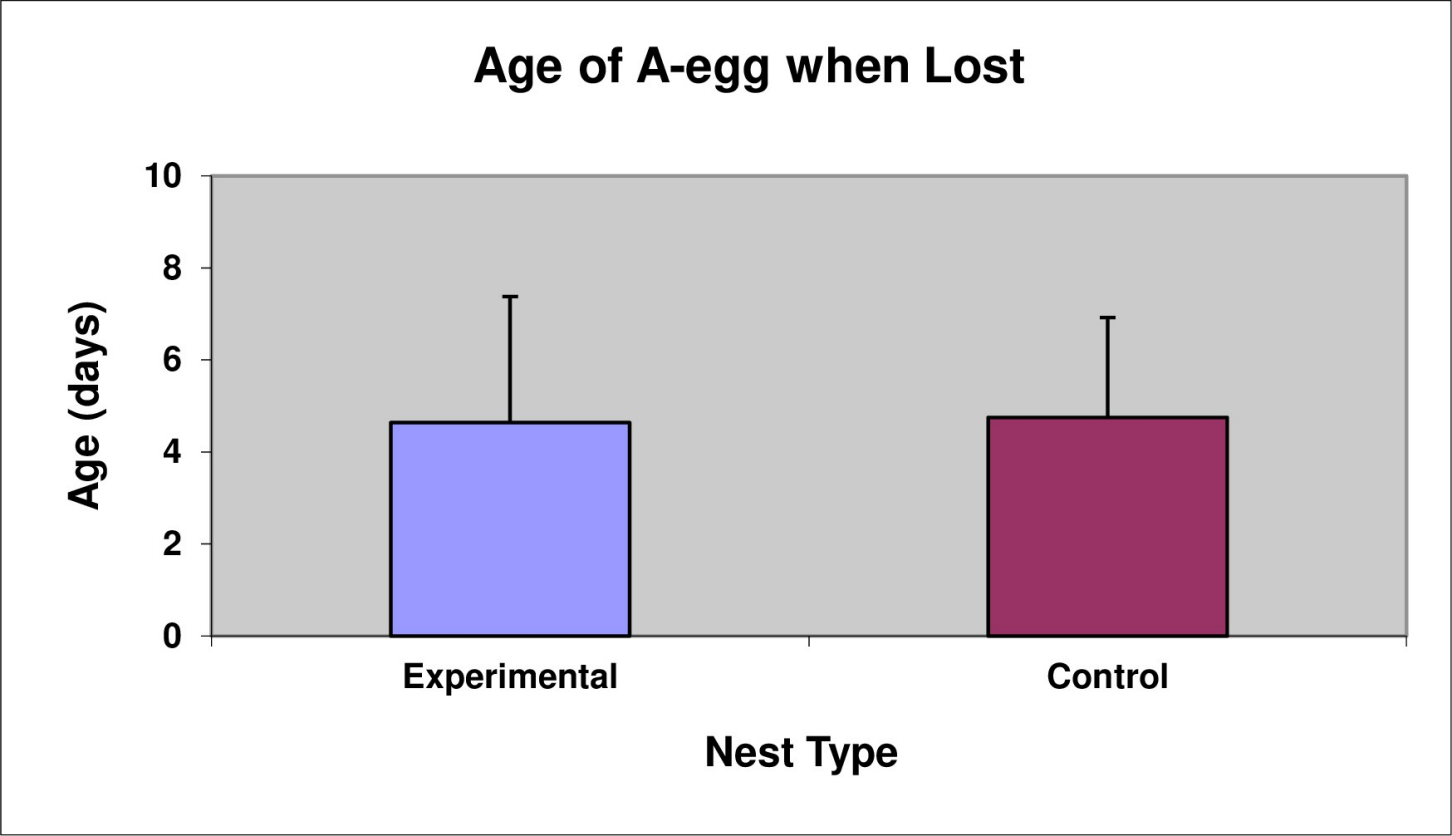
Mean Age in days (± SD) A-eggs were lost in Experimental nests (n = 14) and Control nests (n = 28).

A ring of stones was added around 14 nests in the Experimental Group in order to test whether the longevity of the A-egg could be increased if the A-egg was prevented from being rolled away from the nest either accidentally or deliberately. The Control Group consisted of 28 unmanipulated nests. The ring of stones significantly promoted retention of A-eggs in the nest, with 86% of A-eggs in the Experimental Group being retained in the nest compared to only 4% in the Control Group (Fig 10, Chi-square test, χ^2^(1) = 30.30, P < 0.001). Nevertheless, A-eggs in the Experimental Group did not survive any longer than A-eggs in the Control Group (Fig 11, *t*-test, *t*(40) = -0.14, P = 0.89). In both groups, all the A-eggs were lost.

**Fig 12:**
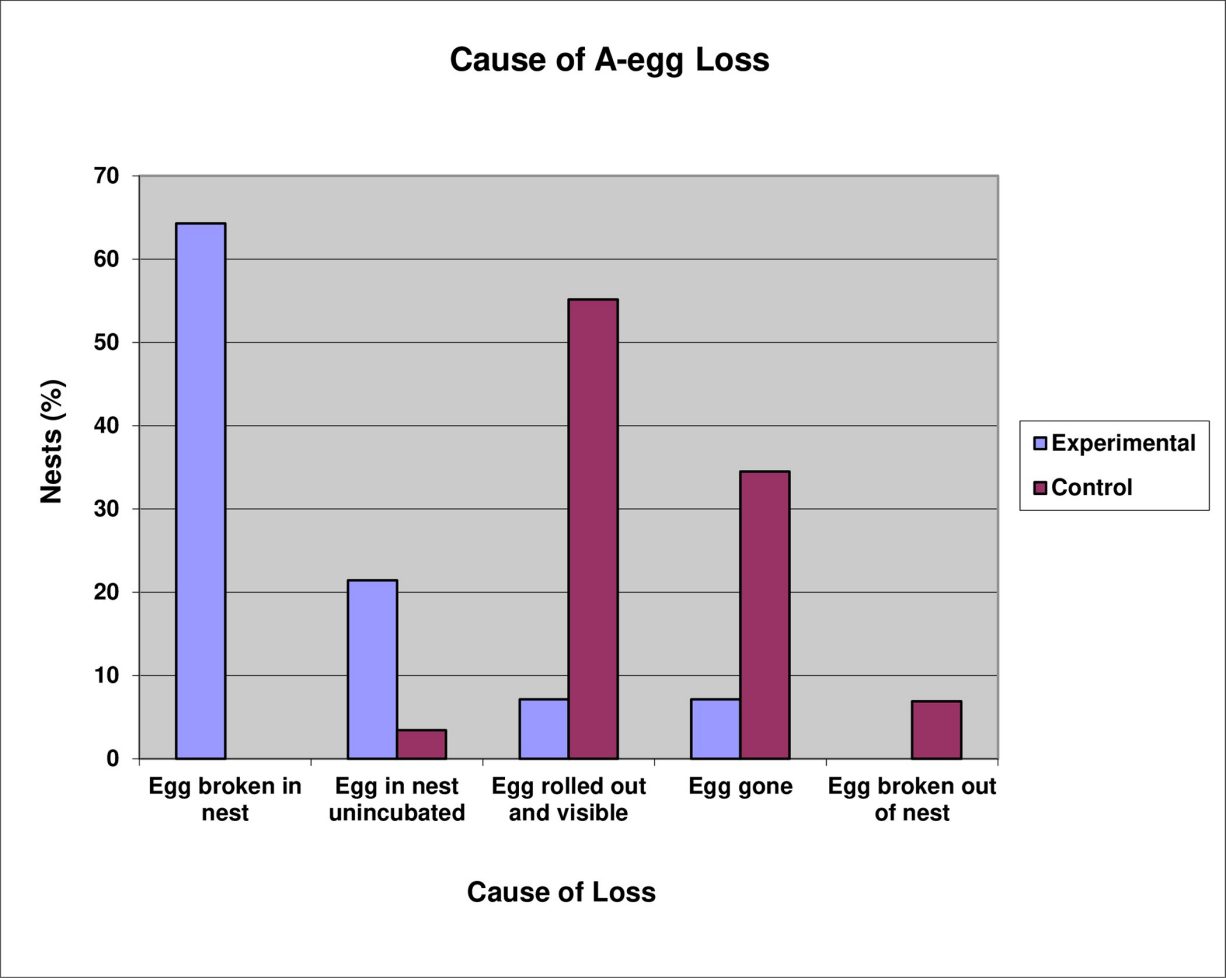
Causes of A-egg losses for Experimental and Control nests.

In the Experimental Group, 64.3% of the A-eggs were broken in the nest, while 21.4% were not incubated even though retained in the nest. By contrast, in the Control Group, the A-egg was found rolled away from the vicinity of 55.2% of nests and had disappeared from a further 34.5% of nests (Fig 12). The results reveal that even when the A-egg remains in the nest, it is still rejected by the incubating parent and perishes at a similar age than had it not been retained in the nest.

### Reproductive hormones

**Fig 13:**
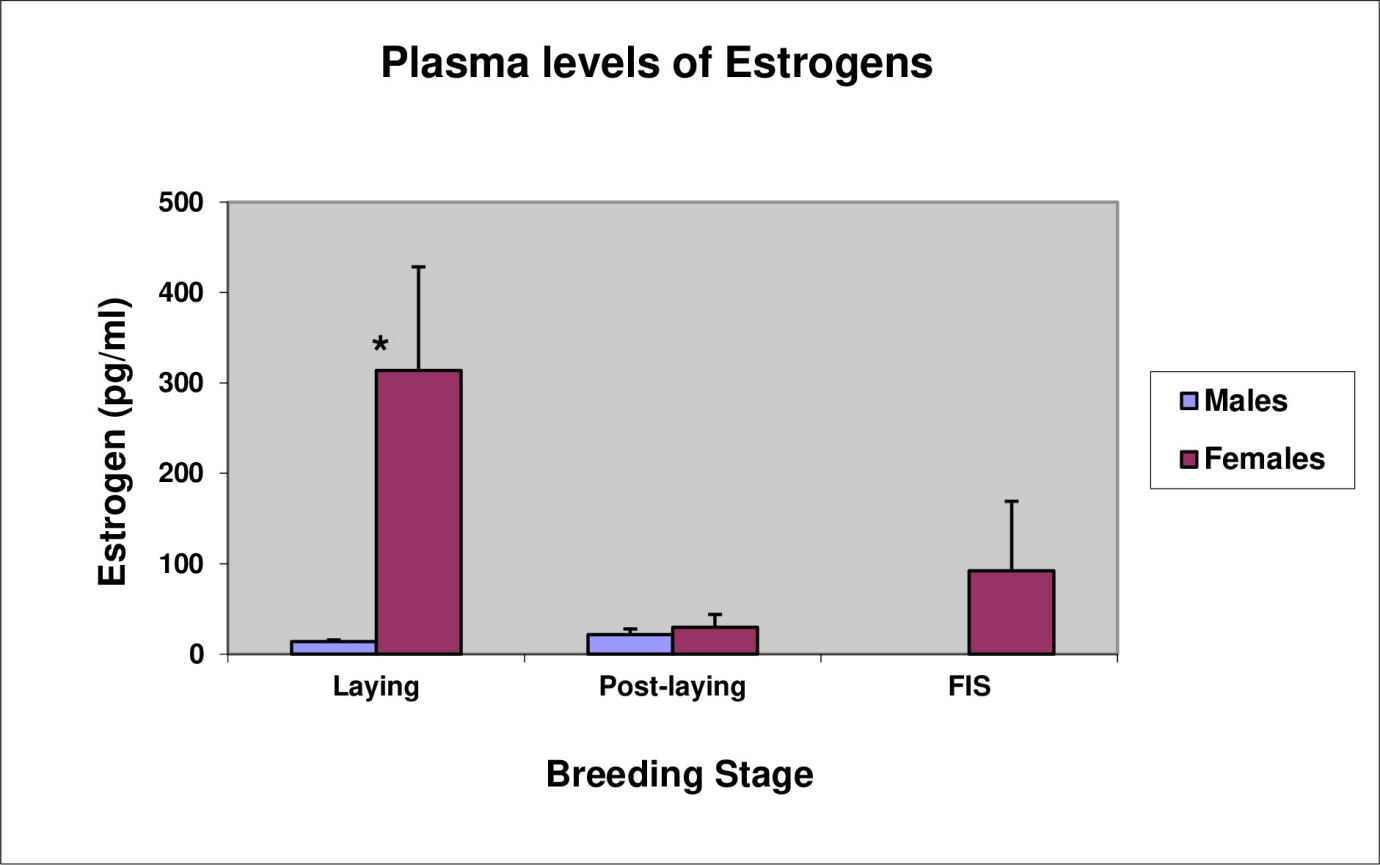
Mean (±se) plasma levels of estrogens (pg/ml) during the laying, post-laying, and first incubation spell (FIS). * indicates that the difference between estrogen levels in males and females is significant (P < 0.05).

As expected, plasma levels of estrogens were significantly higher in females during laying compared to males (*t*-test, *t*(8) = -2.61, P = 0.03), but during the post-laying period, when both sexes were together at the nest site, the basal levels of circulating estrogens were similar for males and females (*t*-test, *t*(8) = -0.52, P = 0.61). Mean plasma levels rose again in females during the first incubation spell when they were incubating alone on the nest (Fig 13).

**Fig 14:**
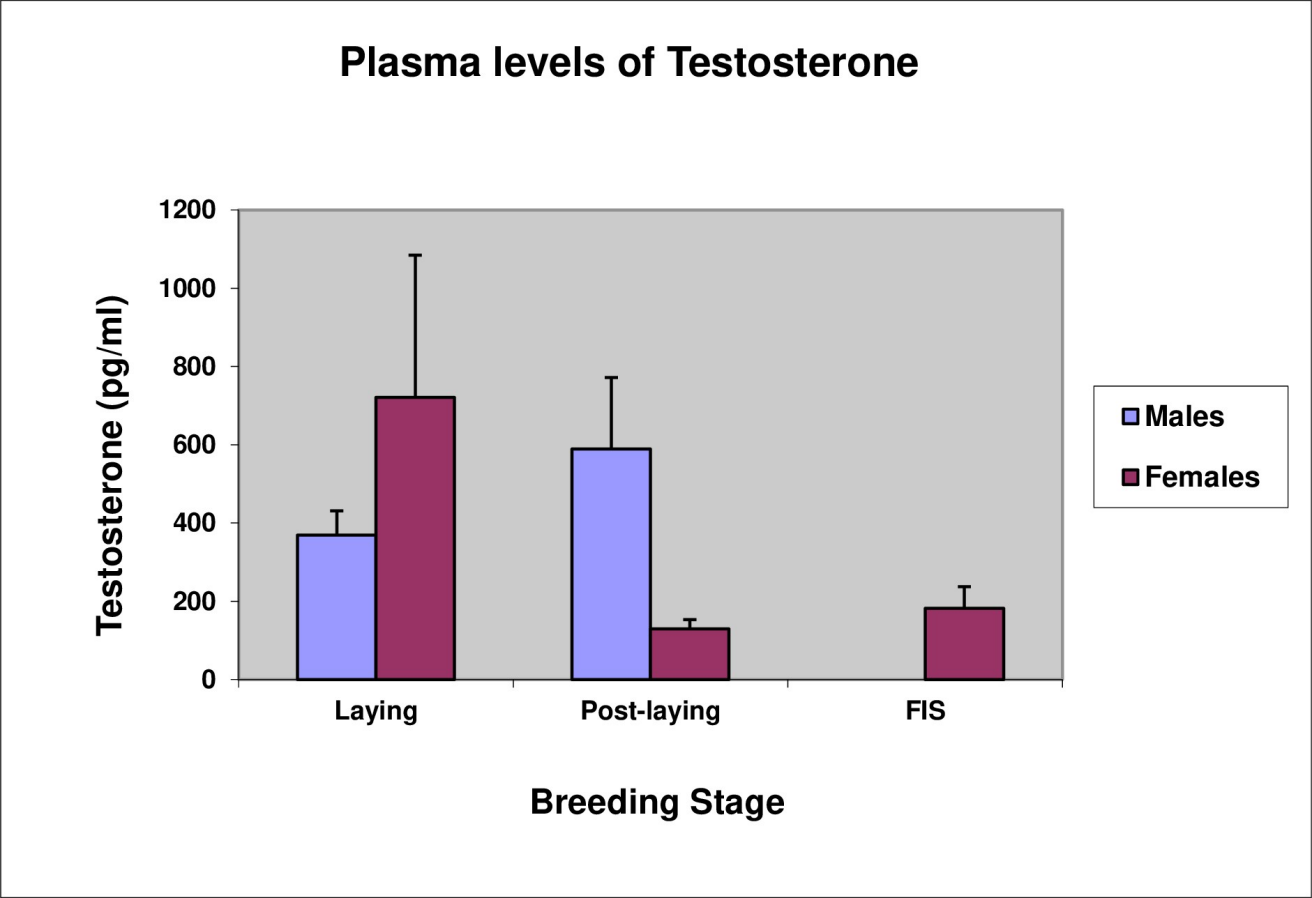
Mean (±se) plasma levels of testosterone (pg/ml) during the laying, post-laying, and first incubation spell (FIS).

By contrast, plasma levels of testosterone did not follow expected patterns. During laying, mean testosterone levels were actually higher in females than males, though the variability amongst females was high and, given the small sample sizes, the apparent difference proved not to be significant (Fig 14, *t*-test, *t*(8) = -0.96, P = 0.37). The results show, however, that circulating testosterone levels in females were at least as high as those in males during the egg-laying period. In the post-laying period, when males remain at the nest to help guard the incubating females, the mean level of testosterone in males was higher than the preceding period, while that in females had dropped, with the consequence that testosterone levels in males were significantly higher than those in females (Fig 14, *t*-test, *t*(10) = 2.49, P = 0.03).

**Fig 15:**
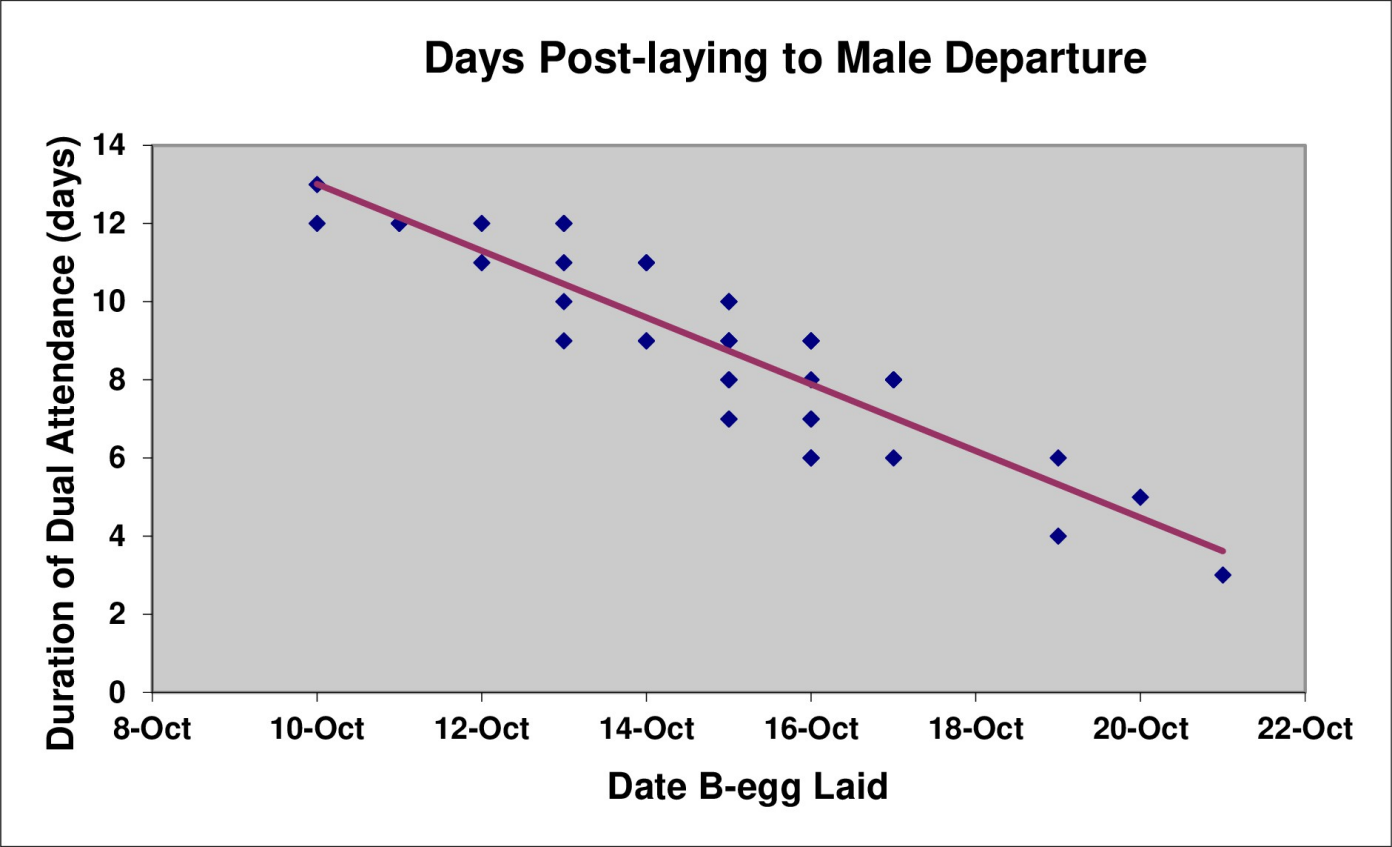
Post-laying attendance by males relative to the date of clutch completion.

This coincided with the period when the male stays at the nest with the female post-laying. The duration the male stays at the nest post-laying was highly negatively correlated (Pearson’s *r*(40) = 0.89, P < 0.001) with the date of clutch completion (Fig 15), with the consequence that the departure of males post-laying happened highly synchronously and the Study Colony virtually emptied out of males over a three-day period from 21–24 October (Fig 1).

### Population census

The total number of erect-crested penguins counted by us on 27–28 October 1998 at five locations on Antipodes Island was 7,510. Compared with a census at the same locations in 1995 [17], the population of all the five colonies decreased, although to different extents (Table 3). Even using the higher count for Orde Lees, the population of breeding erect-crested penguins had reduced by over a quarter in the three years since 1995 (*t*-test: *t*(2) = 5.03, P < 0.05).

**Table 3:** Erect-crested penguin population census.

Location	Mean	Standard error	1995 counts	Change
Alert Bay North	324	7	421	-22.96%
Orde Lees*	4582 (6627)	228 (639)	8720	-47.45% (-24.00%)
Anchorage Bay	1646	18	2779	-40.77%
Stella Bay	303	3	330	-8.08%
Reef Point	654	11	713	-8.27%
**Total**	**7510 (9554)**	**257 (609)**	**12963**	**-42.07% (-26.30%)**

Means (±se) are calculated from the counts of three different observers on the same day.

*Numbers of birds breeding at Orde Lees were also estimated by counting the number of blocks of 70 nesting penguins that fit within the observed area of nesting birds. This count is shown in brackets. Comparisons between the 1998 and 1995 counts [17] are shown.

The accuracy of our counts at Anchorage Bay, Stella Bay and Reef Point was very high. These three locations were also censused in 2011 [2] and 2014 [3] using comparable methods and at a similar time of the year/breeding cycle (Table 4). It is clear that the downward trend in numbers of breeding erect-crested penguins continues, with numbers of active nests of breeding penguins reducing by approximately one-third over a two-decade period in this subsample of the population on Antipodes Island.

**Table 4:** Censuses of active nests of erect-crested penguins in late October at three locations on Antipodes Island over two decades (1995–2014).

Location	1995	1998	2011	2014	Change (%)
Anchorage Bay	2779	1646	2048	1673	-39.8
Stella Bay	330	303	251	222	-32.7
Reef Point	713	654	578	505	-29.2
**Total**	**3822**	**2603**	**2877**	**2400**	**-37.2**

## Discussion

Compared to other penguin species, indeed other species of the genus *Eudyptes*, erect-crested penguins are extreme in the sexual dimorphism of their bills, the dimorphism of their eggs, and the laying interval [42]. Only Macaroni and royal penguins come close, which are the two other crested penguins where obligate brood reduction is so extreme, as none of these species are likely to hatch two chicks, let alone fledge them.

Royal penguins have been observed to deliberately eject the A-gg [43] and, indeed, a similar behavior had been reported for erect-crested penguins [14]. Observations of penguins nesting in our Study Colony were consistent with loss of the small A-egg occurring both accidentally and deliberately. Most often the former occurred when the egg rolled away from the nest, which in the vast majority of cases contained no nesting material at all to prevent the egg from rolling away, or it simply disappeared, having most likely rolled down the slope and off the rock stack on which the colony was situated. Given a clutch of two dimorphic eggs, penguins will preferentially place the larger one in the posterior incubation position against the brood patch, and then attempt to accommodate the smaller egg in the anterior position [44]. The extreme egg-size dimorphism of erect-crested penguins meant that incubating birds, usually females, had difficulty adjusting the small A-egg in the front position of the brood patch. This caused the incubating parent to often sit up and adjust its position, leading to the high proportion of small A-eggs we observed being rolled away from the nest on the day the B-egg was laid. However, our experimental manipulation showed that the A-egg is lost at the same time irrespective of whether it is prevented from rolling away or not, pointing to lack of parental care of the A-egg if not a deliberate rejection of it.

Approximately half of the pairs do little to no incubation of the A-egg prior to laying of the B-egg. Together with very low copulation rates, which are an order of magnitude less than comparably-sized Adelie penguins [45] and low levels of fighting during the courtship period, this points to low levels of reproductive readiness amongst erect-crested penguins arriving at the colony for breeding.

The hormonal levels would seem to support this, with testosterone levels in males being unexpectedly low and in females being unexpectedly high during the laying period. The increase in testosterone in males during the post-laying period when, unlike non-crested penguins, the male remains at the nest with the female after laying, could well be associated with males defending incubating females from “bullying” behavior [30].

### Obligate brood reduction

Our results are not consistent with two of the main hypotheses regarding the reversed egg size dimorphism and obligate brood reduction. First, very few A-eggs are lost to fighting during the courtship period and fighting occurs at very low levels, making it unlikely, as suggested by Johnson et al. [11], that there has been selection to favor the B-egg as a consequence of fighting in the colonies during courtship. Second, given that most A-eggs are lost before or on the day of laying of the B-egg, the A-eggs cannot function as insurance against the loss of the larger B-egg as has been suggested [46].

As Davis and Renner [9] pointed out, these are offshore foraging penguins that probably cannot bring enough food back to the nest to rear two chicks. Erect-crested penguins are derived from ancestors that laid two-egg clutches [9] and, theoretically at least, it would have been possible to reduce clutch size by simply stopping laying another egg after the first one, had selection favored the first-laid A-egg. However, for whatever reason, selection has favored the second-laid B-egg in crested penguins [47]. It being physiologically impossible to have a second egg without laying a first egg [9], the best the crested penguins can do is reduce investment in the first egg. Erect-crested penguins do this to an extreme extent and have the largest egg-size dimorphism of all penguins, indeed, all birds.

The high levels of testosterone we found in females during the laying period may help explain the mechanism for reducing investment. Erect-crested penguins are migratory [5]. In albatross, migratory carryover effects are associated with steroidogenic processes underlying follicle development, such that reproductive success is determined by reproductive readiness associated with differences in steroid hormones [48]. Female albatross that defer breeding have high levels of testosterone. Indeed, Crossin et al. [34] have argued that similar migratory carry-over effects (i.e., a lack of reproductive readiness) apply to Macaroni penguins, which, like erect-crested penguins, have extreme reversed egg size dimorphism and hatch only a single chick. They argue that the small A-egg is, therefore, simply a consequence of a physiological constraint rather than it having an adaptive function. However, this seems too simplistic an explanation, especially given the less extreme reversed egg-size dimorphism seen in other species of crested penguins, which regularly hatch two chicks. High levels of plasma testosterone in females may account for the delay in formation of the brood patch until the B-egg is laid, as observed in Fiordland penguins [47], while higher levels of testosterone deposited in the larger B-eggs may give the chicks hatching from them a competitive advantage against their siblings in species of crested penguins where two chicks normally hatch. Such a mechanism is found in kittiwakes [49].

In sum, the migratory carry-over effect may well explain the mechanism for why crested penguins should invest in the B-egg preferentially as vitellogenesis, which begins in migratory penguins while they are still travelling at sea [34], is constrained until they return to the colony, causing second-laid eggs to be larger. Beyond that, however, delayed incubation through a delay in brood patch development and reproductive readiness, a preference for larger second-laid eggs to be in the posterior incubation position, and higher levels of maternally-deposited yolk androgens are all likely to give chicks from B-eggs a selective advantage [49], favoring the chick from the B-egg in species (i.e., Fiordland, Snares and rockhopper penguins) where both chicks typically hatch. In species of crested penguins with longer foraging distances (i.e., erect-crested, Macaroni and royal penguins), which make it impossible for parents to rear a second chick [9, 42], selection has led to continued reduction in investment in the A-egg. Consistent with these patterns, phylogenetic trees derived from molecular data show that erect-crested, Macaroni and royal penguins are clustered together, while Fiordland, Snares and the rockhopper penguins form a separate genetically closely-related cluster [9].

### Population status

Just as unclear as the evolutionary purpose of the erect-crested penguin’s brood-reduction strategy are its population developments in the past decades. The IUCN red list currently ranks erect-crested penguins as “endangered” due to substantial discrepancy in penguin numbers determined in the late 1970s and counts conducted since the mid-1990s [50].

On the Bounty Islands, penguin numbers until recently were believed to have dropped by more than 75% since 1978 [13, 51–53]. However, a comprehensive drone-based survey in 2019 found that penguin numbers appear to have remained stable, at least since the 1990s. Moreover, comparison of aerial photographs from 1978 and 1998 showed comparable penguin nest numbers, suggesting that the 1978 survey substantially over-estimated numbers of erect-crested penguins [18].

There is similar uncertainty in population estimates of erect-crested penguins on the Antipodes Islands between the late 1970s and the 1990s. Taylor [52] reports on the estimate of 115,000 erect-crested penguin breeding pairs made in 1978, but fails to elaborate on how this estimate was derived. Regardless, this estimate served as a benchmark for the next population survey carried out in 1995 [17] and contributed to the current red list ranking of erect-crested penguins [50]. Yet, if survey data from 1998 onward are compared, the decline of the species on the Antipodes becomes far less clear cut and is heavily weighted by the 1995 count. At Anchorage Bay, there were slightly more erect-crested penguin nests counted in 2014 than during our visit to the island in 1998 (i.e., 1673 vs 1646 nests), while at two other sites, penguin numbers have declined more substantially (Table 4).

These discrepancies highlight the problem with largely opportunistic monitoring of species that are limited to remote locations. More often than not penguin counts are tacked onto expeditions that focus on other conservation or scientific goals and occur sporadically, with large temporal gaps in between subsequent surveys reducing the available data to little more than a series of snapshots that make it difficult to draw any robust conclusions about population trajectories. This is further compounded by the surveys on erect-crested penguins being primarily conducted by New Zealand’s Department of Conservation, which is severely limited in its resources available to carry out this work [54], while at the same time constraining other researchers from visiting New Zealand’s subantarctic islands due to a highly restrictive permitting process [55, 56].

Nevertheless, it seems likely that climate change is negatively impacting erect-crested penguins breeding on the Antipodes Islands. Increased storm frequencies over the past decades likely contributed to an increased occurrence of landslips, especially in the south of Antipodes Island, which wiped out parts of colonies and killed penguins attending their nests [3]. Additionally, there is clear evidence that eastern rockhopper penguins, which also breed on the Antipodes Islands, have experienced a massive population crash since the mid-20^th^ century [57, 58], attributed largely to reduced oceanic productivity [59, 60]. We expect such conditions would affect erect-crested penguins similarly as they are relying on the same ocean region to find food. Yet, it is possible that the larger body size of erect-crested penguins may provide the species with a physiological advantage compared to rockhopper penguins, allowing them to dive longer and deeper [61] and thereby exploit different or shifting prey species. Examining the foraging ecology of erect-crested penguins, about which nothing is known, is likely to be important in order to understand their population trajectory.

However, unless erect-crested penguins receive the scientific attention their current red list ranking demands, the species will remain the enigma of the penguin family.

### Conservation of erect-crested penguins

Remaining an enigma will not change the plight of erect-crested penguins. Research priorities increasingly determine prospects for survival of many species around the world [62]. Although not typically considered to be part of the conservation science toolbox, conservation marketing [63] could be used to influence the outcome for a troubled species like the erect-crested penguin by: (i) summarizing the complex interplay of extrinsic and intrinsic factors that influence the population of erect-crested penguins, (ii) increasing public awareness about this remote penguin species and its potentially precarious situation, and (iii) advocating for increased research efforts and funding by communicating research findings to a wide range of key stakeholders and decision-makers [64]. Without prioritizing conservation marketing for erect-crested penguins, we are likely to be limited, as here, to describing continued declines in the species and, in essence, drafting an obituary for nature [65].
